# Measuring Comorbidity in Cardiovascular Research: A Systematic Review

**DOI:** 10.1155/2013/563246

**Published:** 2013-07-17

**Authors:** Harleah G. Buck, Jabar A. Akbar, Sarah Jingying Zhang, Janet A. Prvu Bettger

**Affiliations:** ^1^School of Nursing, The Pennsylvania State University, 201 Health and Human Development East, University Park, PA 16802, USA; ^2^School of Nursing, Duke University, Duke University Medical Center 3322, 307 Trent Drive, Durham, NC 27710, USA

## Abstract

*Background*. Everything known about the roles, relationships, and repercussions of comorbidity in cardiovascular disease is shaped by how comorbidity is currently measured. *Objectives*. To critically examine how comorbidity is measured in randomized controlled trials or clinical trials and prospective observational studies in acute myocardial infarction (AMI), heart failure (HF), or stroke. *Design*. Systematic review of studies of hospitalized adults from MEDLINE CINAHL, PsychINFO, and ISI Web of Science Social Science databases. At least two reviewers screened and extracted all data. *Results*. From 1432 reviewed abstracts, 26 studies were included (AMI *n* = 8, HF *n* = 11, stroke *n* = 7). Five studies used an instrument to measure comorbidity while the remaining used the presence or absence of an unsubstantiated list of individual diseases. Comorbidity data were obtained from 1–4 different sources with 35% of studies not reporting the source. A year-by-year analysis showed no changes in measurement. *Conclusions*. The measurement of comorbidity remains limited to a list of conditions without stated rationale or standards increasing the likelihood that the true impact is underestimated.

## 1. Introduction

Heart disease and stroke, common cardiovascular diseases, are the third and fourth leading causes of disease burden and the primary causes of death worldwide [1, 2]. Cardiovascular disease (CVD), a systemic disease, rarely occurs alone so it is common to find multiple comorbid conditions in the setting of CVD, particularly in the older adult population who bear a disproportionate share of the comorbidity burden [3]. Comorbidity, at this time, is generally understood to be the presence of other disease entities in the setting of an index disease or condition [4]. However, everything known about the roles, relationships, and repercussions of comorbidity in CVD is shaped by how comorbidity is currently measured. The actual burden of comorbid conditions and the impact on outcomes in CVD may not be fully realized as a result of methodologic limitations in prospective studies completed to date. 

A brief overview of the history of comorbidity measurement will set the stage for understanding these methodologic limitations (Table 1). During the 1970s Kaplan and Feinstein [5] investigated taxonomic problems with classifying comorbidity which they defined as “any distinct additional clinical entity that has existed or that may occur during the clinical course of a patient who has the index disease under study” [6, page 456-7]. According to their conceptualization, comorbidity played one of three roles in relation to the index disease-diagnostic, prognostic, or pathogenic [6]. From this definition and conceptualization they then developed criteria for classifying individuals which could be used by other researchers [5]. Following this early work, Charlson and colleagues [7] in the 1980s developed an instrument, the Charlson Comorbidity Index (CCI), rather than a list of criteria to measure comorbidity. The stated goal of the instrument was to control for sicker individuals in longitudinal clinical trials. The CCI had an advantage of simplicity and ease of use over previous methods, such as Kaplan and Feinstein's [7]. In the 1990s Deyo et al. [8] and D'Hoore et al. [9] each adapted the CCI for use with administrative datasets. During this same timeframe Elixhauser et al. [10] developed a novel measure which defined comorbidity as a “clinical condition that exists before a patient's admission to the hospital, is not related to the principal reason for the hospitalization, and is likely to be a significant factor influencing mortality and resource use in the hospital” (page 10). With this definition Elixhauser clearly delineated that it was the context, hospitalization, not the existence of an index disease, which determined the definition of comorbidity. In all three measures (Deyo, D'Hoore, and Elixhauser) comorbidity was still viewed primarily as a burdensome clinical phenomenon. 

The challenge of managing and measuring comorbidity is gaining increased attention with the worldwide aging of the population [11–14]. In a previous paper published by our group of scientists [15] a systematic review and evolutionary analysis of the use of comorbidity in the empiric literature for adults undergoing care transitions was conducted. The aim of that study was to answer the question as to what was known about the definition, use, and measurement of comorbidity in this at-risk population. However, the lack of robust measurement in almost two thirds of the studies limited what could be stated with any confidence about comorbidity. Attention was drawn to the need for clarity, transparency, and standardization in the measurement of comorbidity in that review. Subsequently, a subgroup was formed to target the measurement of comorbidity in our particular area of expertise—CVD. Specifically, we returned to the original large, comprehensive dataset of studies (*n* = 5,917) and selected out those studies that identified acute myocardial infarction (AMI), heart failure (HF), or stroke as the index disease of the study (*n* = 1432). We then carefully analyzed the measurement of comorbidity in those studies. Our findings are presented in this paper.

Despite more than 30 years of comorbidity measurement, a rigorous systematic review of the measurement of comorbid conditions in CVD outcomes research, particularly in the AMI, HF, or stroke population, has not been conducted and disseminated. Both clinicians and policymakers need to know precisely what is meant by the term comorbidity and how the comorbidity data is measured for two critical reasons—(1) the importance of comorbidity as a descriptor of patient populations; (2) the importance of comorbidity as a potential predictor or modifier of the effect of clinical interventions on outcomes. Imprecise measurement of comorbidities may be creating an incomplete picture of the problem and a misestimation of individual and health system outcomes resulting from unmeasured or mismeasured comorbid conditions in CVD. In addition, variation of comorbidity measurement across studies limits the ability of investigators to aggregate data and conduct meta-analyses necessary for the development of comparative effectiveness research and evidence-based practice protocols. 

Thus, the purpose of this systematic review was to examine the state of the measurement of comorbidity in randomized controlled trials (RCTs) or clinical trials and prospective observational studies of adults hospitalized for an AMI, HF, or stroke. Specifically, we sought to answer four key questions related to the measurement of comorbidity in outcomes research for these three populations: (1) how is comorbidity defined, identified, and measured in studies of acute MI, HF, and stroke? (2) What are the psychometric properties of the measures and indices used? (3) How were the measures used and for what outcomes? (4) Do the definitions, measures, or uses vary by year of publication?

## 2. Methods

### 2.1. Eligibility Criteria

Comorbidity was defined inclusively as any other chronic condition in the presence of AMI, HF, or stroke. To determine the earliest year of publication for inclusion, formal measures of comorbidity were reviewed to identify the year in which commonly used instruments were published (earliest dated to 1969); consequently, articles published in English between 1965 and July 31, 2009, in peer-reviewed journals affiliated with the electronic databases listed below were considered eligible for this systematic review. The search was restricted to randomized controlled trials (RCTs) or clinical trials and prospective observational studies. The rationale for restricting to these types of trials was to exclude studies in which the investigators did not have control over study design related to the measurement of comorbidity as would take place, for example, in retrospective analyses or registry data where existing data is used. If the investigator had control over the measurement of comorbidity (even if the data was obtained from medical records), the study was considered eligible. 

### 2.2. Information Sources

A comprehensive search of the literature was devised and conducted using MEDLINE accessed via PubMed, Cumulative Index of Nursing and Allied Health Literature (CINAHL), PsychINFO, and ISI Web of Science Social Science databases for the original dataset from our previous study [15]. Diverse databases were used to obtain perspectives from multiple disciplines and include both physical and mental health comorbidities. 

### 2.3. Search

Search terms for the original dataset [4, 13, 16] were identified from national reports on comorbidity and concept analyses. Search terms and strategies were developed in consultation with a medical librarian. Although our search strategies were specific to each database due to the options available to customize, our basic search strategy used the National Library of Medicine's Medical Subject Headings (MeSH) key word nomenclature developed for MEDLINE. The exact search strings used in our strategy are given in Appendix/Supplement A (See Supplementary Material available online at http://dx.doi.org/10.1155/2013/563246). The literature search syntax used keywords with the most inclusive suffix. All related terms and combinations of these terms related to the concept of comorbidity (i.e., multimorbidity, co-occurring, coexisting, risk factors, complications, etc.) were used. The literature search for this current analysis was further refined to identify studies including the diagnoses of interest AMI, HF, and stroke as index conditions. 

### 2.4. Study Selection

#### 2.4.1. By Diagnosis

Selected studies were limited to those with adult populations (age ≥ 19 years) hospitalized for an AMI, HF, or stroke. AMI was defined as either ST elevation or non-ST elevation acute MI. Heart failure was defined as an individual having the stated diagnosis on hospital admission (either preserved or reduced systolic function). Stroke was defined as a focal neurologic deficit lasting >24 hours attributed to a cerebral vascular cause of either ischemic stroke or intracerebral hemorrhage or, of shorter duration, a transient ischemic attack. To provide a more homogeneous population for analysis, patients with subarachnoid hemorrhage (often developed secondarily to injury), unstable angina, or symptoms consistent with an acute coronary syndrome (often a preliminary diagnosis) without documented evidence of myocardial ischemia or injury were excluded. Studies reporting populations with mixed CVD diagnoses at enrollment were also excluded. 

#### 2.4.2. By Design

We included randomized controlled trials (RCTs) or clinical trials and prospective observational studies. To be included studies had to report original data with the baseline assessment occurring during the hospitalization and at least one clinical site of multisite studies was to be in the United States. This criterion, once again, provided a more homogenous sample which would facilitate translation of the findings into specific, contextually appropriate recommendations. Retrospective studies designed to use administrative, registry, or public or private claims data were excluded for the reason stated earlier. Secondary analysis, in which the research aim was developed after the dataset existed, was also excluded for the same reason. Meta-analyses, systematic reviews, case reports, editorials, letters to the editor, and pilot studies were also excluded. 

#### 2.4.3. Selection Process

Using prespecified criteria (Appendix/Supplement B), each title and abstract were examined independently by two reviewers for potential relevance. Articles included by any reviewer underwent full-text screening where two independent reviewers read each article to determine if it met eligibility criteria. When the paired reviewers arrived at different decisions about whether to include or exclude an article, they reconciled the difference together with a third-party arbitrator. Articles meeting eligibility criteria advanced to data abstraction. 

We hypothesized that RCTs, in particular, might be less likely to use terms related to comorbidity in the primary outcomes paper (and thus, not be identified by our search). To account for the known prevalence of secondary analysis in CVD trials and to improve the external validity of this systematic review, in a second step we examined the full text article of each RCT identified by an excluded secondary analysis (*n* = 18) from our original search which otherwise met our inclusion criteria. The primary outcomes article and, when published separately, the baseline characteristics or study design articles were identified. These articles advanced to data abstraction as a subanalysis. 

### 2.5. Data Collection Process

The data extraction form was piloted by three investigators with eight studies. Included studies were then abstracted onto the data form by one reviewer and the data confirmed by a second team member. We employed internal quality-monitoring checks through every phase of the project to reduce bias, enhance consistency, and verify accuracy. Examples of internal monitoring procedures were confirmation of study eligibility at each phase (abstract screening, full-text screening, and data abstraction), involvement of two individuals for each level of screening and for data abstraction of each article, and agreement of at least two investigators on all included studies and the data extracted. 

### 2.6. Data Items

Abstracted data elements included first and last author discipline, geographic study location, study design, setting, sample size, patient characteristics (index condition and age), definition of comorbidity, the data source for the comorbidity data, comorbidity measure used, whether the measure was modified from its original use and if so how, stated validity and reliability of the measure, how the comorbidity data or measure was employed in the study, main study outcomes, stated or reviewer-observed limitations of the study related to measurement of comorbidities, and an overview of the study (purpose or question, analytic approach, and main study findings). Conflicting data were resolved by a third reviewer.

### 2.7. Synthesis of Results

Data were summarized across studies to answer key questions 1–3, and then between study variations by index condition, study design and year published were analyzed to answer key question 4. The subanalysis of RCTs identified by excluded secondary analyses focused on key questions 1 and 2 to determine how comorbidities were defined, identified, and measured in this body of literature and the psychometric properties of any indices.

## 3. Results

### 3.1. Study Selection and Characteristics

**Figure 1:**
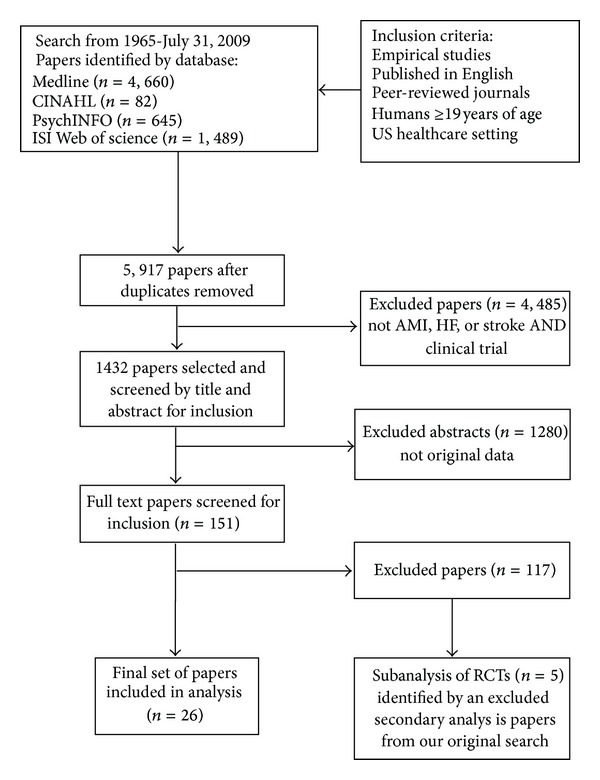
PRISMA flowchart.

Of the 1432 CVD publications reviewed, 26 studies (AMI *n* = 8, HF *n* = 11, stroke *n* = 7) and 5 RCTs (identified by excluded secondary analyses from our original search) met the inclusion criteria (Figure 1) and were analyzed (Table 2). The 26 studies were primarily published in cardiology journals (*n* = 11), by physicians (*n* = 24), and conducted in the USA (*n* = 25). 

### 3.2. Synthesis of Results

#### 3.2.1. Key Question 1: How Is Comorbidity Defined, Identified, and Measured in Studies of Acute MI, HF, and Stroke? 

The following terms were used synonymously to define comorbid conditions: comorbid, concomitant, or underlying diseases or conditions (*n* = 8), risk factors (*n* = 5), relevant clinical variables or data (*n* = 5), patient or clinical characteristics (*n* = 4), past medical history (*n* = 2), or chronic medical conditions or diseases (*n* = 2) in the reviewed studies. The comorbidity data were identified and obtained from 1–4 different sources for a single study including medical records (*n* = 13), clinician judgment (*n* = 8), self/proxy report (*n* = 7), or DSM-III criteria (*n* = 3). In 35% of the studies (*n* = 9) the data source for comorbidities was not reported (Table 3). 

Comorbidities were most commonly recorded, measured, and then analyzed as the presence of individual diseases, conditions, or risk factors (*n* = 21) or laboratory values indicating disease (i.e., lipid levels) without prespecified criteria given for what was or was not counted as comorbidity. The list of individual diseases was unique to each of the 26 studies reviewed. Diabetes was the most frequently measured comorbidity (*n* = 21), followed by hypertension (*n* = 19), dyslipidemia (*n* = 9), and COPD (*n* = 6). Studies generally controlled for cardiovascular diseases other than the index condition. For example, if AMI was the index condition, HF and stroke would be considered comorbidity and controlled for in the analyses. The empirical literature supporting the selection of the conditions and diseases was not cited, and individual conditions defined by some studies as “past medical history” were referred to as “baseline demographics” or “characteristics” in other studies. Definitions of comorbidity or any of the surrogate terms were not provided in any of the reviewed studies. 

The subanalysis of the RCTs identified by excluded secondary analyses (*n* = 18) from our original search revealed that three of these studies used data from primary studies already reviewed in this paper. The 15 remaining secondary analysis papers used data from five clinical trials. Each of these clinical trials used a list of conditions. All five trials (CADILLAC [43], ENRICHD [44], ExTRACT-TIMI 25 [45], GUSTO [46], and VALIANT [47]) identified and analyzed comorbidity as the presence or absence of reported conditions which were identified by laboratory values or preadmission pharmacological therapy. When compared by index diagnosis (e.g., AMI, HF, stroke), the measurement of comorbidities reported in each trial revealed the use of a list of conditions unique to each trial. 

#### 3.2.2. Key Question 2: What Are the Psychometric Properties of the Measures and Indices Used? 

Only five of the 26 studies and one trial in our subanalysis sample reported the use of an established instrument to measure comorbidities [17, 18, 25, 26, 36, 44]. In these five studies and one trial, no evidence was included for the construct validity or reliability (via coefficient alpha) of the instrument for use with the specific study population. All but one study [17] in the overall review (*n* = 25) had sufficient sample size to provide for the assessment of these statistics to allow for greater confidence in the interpretation of the results from the instruments. 

#### 3.2.3. Key Question 3: How Were the Measures Used and for What Outcomes? 

Three studies examined only medical comorbidities using the CCI. Chin and Goldman [25] used the CCI as a summary measure of coexisting diseases to identify predictors of readmission or death for patients admitted to the hospital with shortness of breath, fatigue, or HF. Rocha and colleagues [17], with Charlson as a coauthor, used the CCI to assess medical comorbidity in a study that evaluated potential predictors of posttraumatic stress disorder in AMI. The ENRICHD trial [44] tested an intervention for treating depression and low perceived social support after AMI. The mean CCI score was reported as a medical characteristic in the primary outcomes paper of the ENRICHD trial but not the baseline characteristics paper or secondary analysis that was identified in our search. The primary outcomes paper explained the CCI's use only as a footnote in the demographics table. 

Two studies examined comorbid depression using different instruments, while a third study examined depression plus other medical conditions. Kishi and colleagues [36] assessed comorbid depression in stroke patients using the Hamilton Depression Rating Scale. Romanelli et al. [18] measured comorbid depression in older adults with AMI using the Beck Depression Inventory. Fulop et al. [26] examined both comorbid depression and medical conditions separately using the Duke Severity of Illness Checklist to derive both an overall disease severity score and then an individual comorbidity score to control the effects of severity of illness on medical resource use in older adults with HF while also examining comorbid depression as measured by the Geriatric Depression Scale. 

Comorbidity was used in multiple ways in the analyses of the reviewed studies (Table 3). The majority of the studies (*n* = 20) used comorbidity as a covariate. However, comorbidity was also used as a predictor (*n* = 7), outcome (*n* = 3), and descriptor (*n* = 2). Reported outcomes of comorbidity were increased morbidity (*n* = 12), health service utilization (*n* = 7), mortality (*n* = 7), and quality of life (*n* = 3). 

#### 3.2.4. Key Question 4: Do the Definitions, Measures, or Uses Vary by Year of Publication?

A year-by-year analysis of the studies showed no changes in definitions or measurement over the 14 years of publication. No pattern of improving definition and operationalization of the variable, such as standardization or a theoretically derived definition, or greater use of validated instruments was found when the studies were analyzed by year or decade or when they were examined by study design.

## 4. Discussion

### 4.1. Summary of Evidence

The purpose of this systematic review was to examine the measurement of comorbidity in CVD clinical trials. We identified that 21 out of 26 studies measured comorbidities with a list of unsubstantiated diseases without defining comorbidity while using multiple data sources or leaving the data source unknown. This is particularly troubling given the multiple valid and reliable measures available to researchers [11, 48]. In the small number of studies utilizing an instrument, the CCI was the most frequently used. Equally troubling was the finding that measurement did not change or advance during a time period when the methodology of CVD trials was improving overall [49]. In the following section, we will discuss the two challenges experienced in conducting this systematic review followed by the three potential threats to the validity of comorbidity measurement that we identified.

### 4.2. Two Challenges in Conducting a Review of Comorbidity Measurement in CVD Trials

A major challenge in conducting this review arose from the a priori decision to use only prospectively collected data. While this decision was made to capture studies where the investigators designed the study and therefore determined how comorbidity was measured, this resulted in a surprisingly low number of studies. Thus, while we started with a relatively robust pool (*n* = 1432), resulting in 151 studies for full screening, when all mixed cohort populations, registry studies, and secondary analysis or retrospective studies were excluded the final set of studies was a relatively small sample of 26. We addressed this challenge by then adding a subanalysis of the methods papers of the RCTs identified (but excluded) as secondary analyses. This particular challenge highlights that many published CVD papers are derived from previously collected data for which the measurement of comorbidity was never the main outcome of interest. If the validity of the comorbidity data in the parent trial suffers from internal threats, these threats carry over into all subsequent secondary analyses and what is known or knowable about comorbidity suffers. This systematic review draws attention to the critical need to strengthen the measurement of comorbidity in future multinational CVD clinical trials so that the relationships between comorbidity and outcomes can be trusted. The trustworthiness of these relationships becomes especially important when translating intervention studies into clinical practice for more heterogeneous populations.

A second challenge was the lack of a single, logically coherent, definition of comorbidity in CVD research. Despite constructing an electronic literature search using related terms (*n* = 27) identified from national reports and concept analyses on the topic of comorbidities [15], it is likely that studies of adults hospitalized for AMI, stroke, or HF were not identified. In addition, focusing on clinical trials, with their known exclusion of the complex chronically ill, may have introduced bias in the findings. The exclusion of meta-analyses, because of their potential for including studies already captured in the systematic search, may have excluded equally valid, but unincluded, studies. Adding to this challenge there was a lack of salient information about comorbidity and its measurement in the studies that we did identify and review. We hypothesize that manuscript length restrictions in the particular studies that we reviewed could have constrained the capacity of authors to fully describe each variable and source of information. Our review of the methodology papers from the subanalysis of the larger clinical trials supported this, when little to no information was found regarding rationales for particular conditions measured or the data source on the conditions. Or it is possible that among clinicians the definitions of risk factor or clinical characteristics may be assumed common knowledge needing no further explanation. However, this review showed that despite the paucity of information on the measurement of comorbidity, what could be determined was that each study uniquely operationalized the term comorbidity and how it was employed statistically. 

### 4.3. Three Potential Threats to the Validity of Comorbidity Measurement

#### 4.3.1. Heterogeneity in Data Sources

Heterogeneity in the comorbidity data or *what* is measured was noted in a recent systematic review of multimorbidity instruments which found that 59% of the studies analyzed used a list of diseases without any indication of the rationale behind the selection of the specific conditions [11]. Our analysis of CVD studies supports this finding but highlights an additional potential threat in what is measured—the lack of definitions for what is considered comorbidity. The selection of conditions without a stated rationale, as we found, presents an internal threat to the validity of the study by potentially introducing investigator bias. For example, important confounding conditions could be excluded because investigators have not traditionally measured them. The threat is increased when data for these comorbidity lists come from multiple sources such as patient or proxy self-report, clinician assessment, and medical records as was also found in this review. Concordance between different data sources in comorbidity is known to be problematic [50]; however, the use of a validated index does not assure freedom from internal threats to validity. The two studies in our review that used the CCI appear to have accrued the information from chart review [25] or clinician administered interview [17]—two very different data sources making comparisons of their findings problematic. Further heterogeneity in the data arises from the use of administrative datasets as noted earlier [51]. The Elixhauser measure and Deyo and D'Hoore's adaptation of the CCI all depend on International Statistical Classification of Diseases (ICD) codes. Use of ICD codes has such problems as the known underreporting of comorbidities [9], difficulties in distinguishing comorbidities from complications of treatment or severity indicators for the index disease [10], code selection associated with better payment [8], and database-specific limitations (e.g., number of comorbidities allowed by the database) [9]. But despite these limitations, each comorbidity measure has been shown to strongly predict complications, functional decline, and death in hospitalized adults [7–10]. 

In recognizing these limitations, investigators leading prospective studies have the opportunity to design the measurement of comorbidities to reduce the likelihood of heterogeneity with its missingness and inaccuracies. Recognizing that 50% of the studies in this review relied on medical records as a data source for comorbidity, it might be informative to review and synthesize how comorbidity is measured among studies relying on medical record data accrued for clinical practice rather than research. Whether the use of clinical data versus research data results in differences in prediction of patient outcomes is an interesting and needed line of inquiry. 

#### 4.3.2. Variability in Measurement

Variability in measurement practices or *how* comorbidity is measured presents the second potential threat to the validity of comorbidity measurement in CVD trials. A national report on comorbidities highlighted that the definition and measurement of comorbidity in clinical trials is known to vary based on the aims and outcomes of the studies [4]. Comorbidity is gaining increasing attention while its measurement, as documented in this review, apparently remains static over time and without standards for parameters. Past and current trends in measurement may reflect mentoring networks and research training rather than best practice (e.g., measurement is guided by who is part of the research team and how they were trained). This variability presents several threats. For example, in one HF study reviewed [25] the investigators used the CCI to measure comorbidity while also controlling for HTN, creatinine, and DM (all measured in the CCI) individually in some of their analyses. This leads to potentially weighting or confounding in the analysis and makes assessing the outcomes problematic. In observational studies, the prevalence of particular comorbid conditions may be under (or over) estimated or under (or over) reported, and their influence on outcomes unknown if this is a routine practice. When comparing clinical trials with similar aims and outcomes, an accurate estimate of the effect size may be difficult to assess if comorbidities were measured differently in different intervention studies. Meta-analyses may be unknowingly amalgamating vastly different populations because of variability in the measurement. This paper confirms this documented variability in measurement. 

#### 4.3.3. Meaningfulness of the Findings

The third potential threat to the validity of comorbidity has direct patient care implications. Even if the measurement of comorbidity was to improve significantly, the impact of this to the individual patient is unclear. While we may be able to report the amount of variance that a particular disease accounts for in a particular outcome, as to whether that is meaningful to the individual patient is unknown and perhaps currently unknowable. Furthermore, there may be unknown, unmeasured confounders that would come to light when patients' chronic illness experiences are carefully explored. For example, by exploring the patient experience of comorbidity we might discover why patient nonadherence to treatment rates in chronic illness (comorbidity) has stayed fairly stable over several decades at approximately 50% [52]. This lack of empiric studies into the patient comorbidity experience was noted by our group in an earlier conceptual study [15]. By continuing to measure comorbidity as we always have we derive no new knowledge that might lead to improvements in patient care. 

## 5. Conclusion

 This systematic review suggested that the burden of comorbidity for individuals with CVD may not be fully realized as a result of methodologic limitations in the prospective studies we reviewed. CVD outcomes research would benefit from the development of a standard definition and standard measures that all studies could use. Furthermore, research is needed into how to best capture and measure patient-reported experiences with comorbid conditions. We recommend that future studies be designed using valid and reliable indices or appropriate or theoretically chosen comorbidities when indices are not appropriate and transparency in all studies by providing the rationale and limitations for one approach to measuring comorbidities over another. 

## Supplementary Material

The literature search syntax, abstract screening guidelines, and PRISMA checklist for this systematic review are available. Use of the search terms in Appendix A in MEDLINE accessed via PubMed, Cumulative Index of Nursing and Allied Health Literature (CINAHL), PsychINFO and ISI Web of Science Social Science databases should reproduce our search. Application of the abstract screening guidelines (Appendix B) to the abstracts generated by the search should reproduce our final set of papers included in the analysis. The Preferred Reporting Items for Systematic Reviews and Meta-Analyses (PRISMA) checklist provides evidence for the transparent and complete reporting of this systematic review.

## Figures and Tables

**Table 1:** Historical overview of the measurement of comorbidity.

	Kaplan and Feinstein [5]	Charlson et al. [7]	Deyo et al. [8]	D'Hoore et al. [9]	Elixhauser et al. [10]
Time frame	1970s	1980s	1990s	1990s	1990s

Purpose	Classify patients for therapeutic and statistical reasons	Prospectively identify persons at greater risk of death from comorbid diseases	Adapted the CCI for use with administrative datasets	Adapted the CCI for use with administrative datasets	Predict resource use or clinical outcomes

Original population	Diabetics	Medical patients Female breast cancer patients	Medicare lumbar spinal surgery patients	Hospitalized patients in Quebec, CAN	Acute care patients in CA

Measurement method	Clinician derived from symptom patterns, disease duration, physical exam, and lab tests	Clinician scored from list of weighted diseases Validated against Kaplan and Feinstein [5]	Used ICD-9-CM codes equivalent to diseases in the CCI	Implemented an algorithm to map the ICD-9 codes to CCI components	Developed a set of 30 comorbidities with their ICD-9-CM codes

Predictors of comorbidity	Clinical (e.g., vascular or nonvascular diseases) variables	Sociodemographic and clinical variables	Sociodemographic variables and clinical variables	Sociodemographic and clinical variables	Sociodemographic and clinical variables

Outcomes assessed	Mortality or vascular complications for those patients who survived	Mortality	Mortality, hospital complications and treatments, discharge destinations	Inpatient mortality	Mortality and fiscal

Surrogate terms for comorbidity	Episodic events, disease, ailment, and chronic condition	Common conditions	Chronic conditions	Complications (if iatrogenic)	Clinical condition, preexisting condition

CCI: Charlson Comorbidity Index, ICD: International Statistical Classification of Diseases.

**Table 2:** Demographics of the studies by diagnosis.

	AMI	HF	Stroke
Number of studies	8 [17–24]	11 [25–35]	7 [36–42]

Publication years	1997 [22]–2008 [17, 20]	1997 [25]–2009 [35]	1995 [38]–2009 [42]

Journal type	Cardiac [20, 22, 24], nephrology [19], general medicine [21], nursing [23], psychology [17], and geriatric [18]	Cardiac [25, 29–32, 34, 35], psychology [26, 33], general medicine [27], and geriatric [28].	Neurology/stroke [36, 39–42], cardiology [37], and psychology [38]

Author discipline	Medicine [18, 19, 21, 24], medicine plus another discipline [17, 20, 22], or nursing [23]	Medicine [25–27, 29–32, 34, 35], medicine plus another discipline [33], or nursing [28]	Medicine [36–42]

Country of study	USA except one [22]	USA	USA

Study question related to comorbidity	3 [17, 19, 21]	4 [25, 26, 31, 33]	3 [36, 38, 40]

Used a comorbidity instrument	CCI [17]	CCI [25], Duke Severity of Illness Checklist [26], Geriatric Depression Scale [26], and Beck Depression Inventory [31, 33]	Hamilton Depression Rating Scale [36]

Main outcomes	Mortality [18, 19, 21, 22], morbidity [20, 21, 23, 24], and disability [17]	Mortality [25, 27, 31], disability [26, 28, 33], QoL [28], and health service utilization [25, 28–30, 32, 34]	Morbidity [38–40, 42], QoL [37, 38], and health service utilization [41]

CCI: Charlson Comorbidity Index, QoL: quality of life.

**Table 3:** Studies in final analysis.

Investigators	Index condition	Sample	Definition of comorbidity *Surrogate terms *	Diseases listed **(instrument)**	Data source	Comorbidity used as
Afshinnia et al. [19]	AMI	*n* = 220	No definition *comorbid diseases, conditions, underlying diseases *	HTN, DM, HF, sepsis, anemia, cardiorespiratory arrest	Patient, family Clinician judgment Medical records	Covariate

Afzal et al. [34]	HF	*n* = 163	No definition *comorbid conditions, risk factors *	HTN, DM, Hx of MI/stroke	Clinician judgment Medical records	Covariate

Ariyarajah et al. [37]	Stroke	*n* = 66	No definition *common medical comorbidities* *risk factors *	Hx of stroke, Afib CAD, MS, MR, dilated, restrictive, and hypertrophic cardiomyopathy, hyperlipidemia, DM, hyper/hypothyroid, COPD, HF, MI	Medical records	Covariate

Castillo et al. [38]	Stroke	*n* = 142	No definition *comorbid depression *	Depression	Clinical judgment using DSM-III criteria	Predictor interaction term

Chin and Goldman [25]	HF	*n* = 257	No definition	Hx of HF, MI, HTN, DM **(Charlson Comorbidity Index)**	Medical records	Predictor and covariate

Freedland et al. [33]	HF	*n* = 613	No definition *comorbid medical condition *	Hx of HF, MI, anemia, arthritis, CAD, CVA, DM, GI disorder, HTN, COPD, sleep apnea, renal disease Hx of 1 or more comorbid medical conditions **(Beck Depression Inventory)**	Clinical judgment using the diagnostic interview schedule Medical records	Predictor, covariate, outcome

Fulop et al. [26]	HF	*n* = 203	No definition	**(Geriatric Depression Scale;** **Duke University severity of illness checklist)**	Patient Clinician judgment Medical records	Covariate

Goonewardena et al. [32]	HF	*n* = 75	No definition	HTN, DM, COPD, CKD, Afib, depression	Unclear	Covariate

Jiang et al. [31]	HF	*n* = 1,006	No definition *concomitant illnesses, clinical characteristics *	Hx MI, DM	Medical records	Covariate

Kimmelstiel et al. [30]	HF	*n* = 200	No definition	HTN, DM	Patient Medical records	Covariate

Kishi et al. [36]	Stroke	*n* = 301	No definition	**(Hamilton Depression Rating Scale)**	Patient Clinician judgment using the DSM-III criteria	Predictor

Sert Kuniyoshi et al. [20]	AMI	*n* = 92	No definition *characteristics *	HTN, DM, HF hypercholesterolemia	Unclear	Covariate

Lakkireddy et al. [21]	AMI	*n* = 376	No definition *characteristics *	HTN, Hx MI, DM hypercholesterolemia	Unclear	Predictor covariate

Malki et al. [29]	HF	*n* = 187	No definition	HTN, DM, Hx MI, stroke	Clinician judgment	Covariate

Marrugat et al. [22]	AMI	*n* = 1460	No definition *clinical variables *	CKD, COPD, DM, HTN, PVD	Unclear	Covariate

Mehta et al. [39]	Stroke	*n* = 80	No definition *clinical data, other diseases, risk factors *	HTN, DM, CHD, dyslipidemia	Unclear	Covariate

Moroney et al. [40]	Stroke	*n* = 185	No definition *risk factor *	Angina, MI, HF, and valvular heart disease	Patient Family Key informants Medical record	Covariate

Naylor et al. [28]	HF	*n* = 239	No definition *medical conditions, health conditions *	CAD, HTN, Afib, DM, pulmonary disease	Medical record	Covariate

Quinn [23]	AMI	*n* = 100	No definition *disease history* *clinical variables* *past medical history *	Hx of Angina, CAD, HTN, DM, hyperlipidemia, smoking (past/current), previous MI	Medical record	Covariate

Rehan et al. [24]	AMI	*n* = 92	No definition *baseline demographics *	Hx of CAD, HTN, HF, DM	Unclear	Descriptor

Rocha et al. [17]	AMI	*n* = 25	No definition	(**Charlson Comorbidity Index ** for medical comorbidities; **SCID and IES-R** for PTSD)	Patient Medical record	Predictor, outcome

Romanelli et al. [18]	AMI	*n* = 153	No definition	Depression DM, COPD, HTN, hyperlipidemia, CKD, **(Beck Depression Index)**	Patient Clinician judgment using the DSM-III Medical record	Predictor, outcome

Sakr et al. [27]	HF	*n* = 34	No definition *risk factor *	CAD, CKD, pneumonia, DM, HTN, HF	Unclear	Covariate

Shah et al. [41]	Stroke	*n* = 81	No definition *clinical data *	DM, HTN, hypercholesterolemia, CAD, Afib, Hx of stroke	Unclear	Covariate

Soman et al. [35]	HF	*n* = 201	No definition *relevant clinical variables *	CAD, Hx of MI DM, HTN, lipid abnormalities	Clinician judgment	Covariate

Stead et al. [42]	Stroke	*n* = 418	No definition	HTN, DM, Afib, Hx of TIA, and stroke	Unclear	Descriptor

AMI: acute myocardial infarction, Afib: atrial fibrillation, CAD: coronary artery disease, CHD: chronic heart disease, CKD: chronic kidney disease, COPD: chronic obstructive pulmonary disease, CVA: cerebral vascular accident, DM: diabetes mellitus, DSM-III: American psychiatric association diagnostic and statistical manual of mental disorders, 3rd edition, GI: gastrointestinal, HF: heart failure, HTN: hypertension, Hx: history, IES-R: impact of event scale-revised, MI: myocardial infarction, MS: mitral stenosis, MR: mitral regurgitation, PVD: peripheral vascular disease, SCID: structured clinical interview for DSM disorders, TIA: transient ischemic attack.

Diseases listed are in regular font. Instruments used in the study are in bold.
